# Preliminary Identification of Key Genes Controlling Peach Pollen Fertility Using Genome-Wide Association Study

**DOI:** 10.3390/plants10020242

**Published:** 2021-01-27

**Authors:** Zhenyu Huang, Fei Shen, Yuling Chen, Ke Cao, Lirong Wang

**Affiliations:** 1 Zhengzhou Fruit Research Institute, Chinese Academy of Agricultural Science, Zhengzhou 450009, Henan, China; huangzhenyu@caas.cn (Z.H.); chenyuling@caas.cn (Y.C.); 2 Beijing Agro-biotechnology Research Center, Beijing Academy of Agriculture and Forestry Sciences, Beijing 100097, China; shenf1028@gmail.com

**Keywords:** *Prunus persica*, pollen fertility, GWAS, candidate gene identification, ABCG transporter

## Abstract

Previous genetic mapping helped detect a ~7.52 Mb putative genomic region for the pollen fertility trait on peach Chromosome 06 (Chr.06), which was too long for candidate gene characterization. In this study, using the whole-genome re-sequencing data of 201 peach accessions, we performed a genome-wide association study to identify key genes related to peach pollen fertility trait. The significant association peak was detected at Chr.06: 2,116,368 bp, which was in accordance with the previous genetic mapping results, but displayed largely improved precision, allowing for the identification of nine candidate genes. Among these candidates, gene *PpABCG26*, encoding an ATP-binding cassette G (ABCG) transporter and harboring the most significantly associated SNP (Single Nucleotide Polymorphism) marker in its coding region, was hypothesized to control peach pollen fertility/sterility based on the results of gene function comparison, gene relative expression, and nucleotide sequence analysis. The obtained results will help us to understand the genetic basis of peach pollen fertility trait, and to discover applicable markers for pre-selection in peach.

## 1. Background

Peach (*Prunus persica* (L.) Batsch) is one of the most important fruit crops worldwide, possessing extensive genetic diversity and high economic value. Male sterility, manifesting as an abnormally developed stamen with no fertile pollen generated, is a widespread phenomenon observed in flowering plants such as peach [1]. Due to pollen abortion in peach cultivars such as ‘Chinese Cling’, ‘Annong Shui Mi’, ‘Kurakato Wase’, ‘Wan Huang Jin’, ‘Da Duan Mi Lu’, and ‘Shenzhou Shui Mi’, proper pollinating trees must be planted to maintain stable fruit yields [2]. However, natural cross-pollination is easily affected by undesirable climate conditions, while manual pollination is laborious and inefficient, thus making pollen fertility a key breeding objective in peach.

Pollen fertility is closely related to the tapetum, which is the innermost layer of the anther wall, and is a secretory cell layer that functions as the source for the synthesis and transport of nutritional and structural molecules for pollen wall formation [3]. The tapetum undergoes degradation induced by programmed cell death (PCD) during meiosis, and immature or delayed tapetum PCD usually affects pollen development and male sterility [4,5]. In the past few years, several candidate genes reasonably associated with pollen fertility have been successfully identified in model plants, such as *Arabidopsis* and rice, and these genes mainly involved in three functions: (1) tapetum development (*Udt1* [6], *TDR* [4], *MS1* [7], *DYT1* [8], *TDF1* [9]); (2) biosynthesis and modification of various lipidic precursors for pollen wall formation in the tapetum (*MS2* [10], *CYP703A* and *CYP704B* [11,12], *ACOS* [13], *PKS* [14], *AMS* and *MS188* [15]); (3) the allocation of compositions from the tapetum to the anther locule for pollen wall formation (ATP-binding cassette G (*ABCG*) transporter-encoded genes [16,17,18,19,20]).

As for peach, even though the key factors controlling pollen fertility remain unreported, attempts have been made to discover candidate genes through forward genetic approaches. It has long been known that pollen sterility is determined by a single gene pair, *Ps/ps*, with the pollen fertile allele completely dominant over the pollen sterile allele [21], and these results have been confirmed in studies on inbred F_1_ progeny of ‘Okubo’ [22], and crossbred F_1_ populations of ‘91-42-51’ × ‘Ruiguang 2’ [23] and ‘Yumyeong’ × ‘Baekhyang’ [24]. The *Ps* locus was first reported to be located between a codominant RAPD (Random Amplified Polymorphic DNA) marker, Q40cod, co-segregating with AC-CAT3 (4 cM), and two co-segregating AFLP (Amplified Fragment Length Polymorphism) markers, AA-CAT4 and ACA-CAT3 (3 cM), on peach linkage group (LG) 08 (corresponding to LG 06, according to the follow-up study) using a genetic map of ‘Ferjalou Jalousia’ × ‘Fantasia’ population [25]. Another RAPD marker, UBC405_2300_, has been verified as co-segregated with the *Ps* gene in 115 individuals of the ‘Yumyeong’ × ‘Baekhyang’ population; the recombination frequency between this marker and the *Ps*/*ps* locus is 4.3%, and this marker has been found to be generally adequate to identify the *Ps* allele in other segregating progeny and commercial cultivars [24]. In the ‘Ferjalou Jalousia’ × ‘Fantasia’ F_2_ population, the *Ps* gene is located on the top of LG 06, and the nearest marker is FG40 at 4.8 cM [26]. Later, by screening 122 SSR (Simple Sequence Repeats) markers in 637 peach cultivars and 138 individuals of the ‘Ruiguang 19’ × ‘Summergrand’ F_1_ population, two markers, CPDCT013 and CPSCT012, on the top of LG 06, were proven to be linked tightly with the *Ps* locus, being mapped at 18.9 cM and 39.8 cM, respectively [27]. Taken together, the top region of peach LG 06 has proven to be a hotspot harboring key candidate genes underlying the pollen fertility trait.

As an alternative approach to conventional genetic mapping, the genome-wide association study (GWAS) has rapidly come into focus for the genetic dissection of complex traits by exploiting the linkage disequilibrium (LD) present among individuals from natural populations or germplasm collections, offering advantages including increased resolution, a reduced research period and improved allele number detection [28,29]. Several comprehensive GWAS projects have shed light on the genetic basis of important agronomic traits, such as fruit size and shape, fruit sugar and acid content, fruit texture, and chilling requirement, and have provided us with reliable markers for efficient genomic selection [30,31,32,33,34,35,36].

In the present study, we performed a large-scale GWAS using re-sequencing data generated from 201 peach accessions, aiming to identify key candidate genes controlling the pollen fertility trait, and discover applicable markers for pre-selection in peach.

## 2. Results

### 2.1. Phenotyping

**Figure 1:**
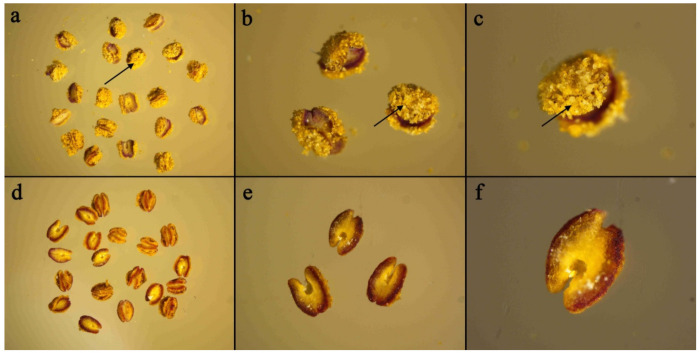
The anther observation of the peach fertile/sterile phenotype under different magnifications. The black arrows indicate the mature pollen grains. (**a**–**c**) indicate the fertile phenotype with abundant pollen grains in the naturally dried anthers. (**d**–**f**) indicate the sterile phenotype with no pollen grains observed.

By observing naturally dried anthers, a total of 180 peach accessions with abundant pollen grains were classified as fertile (Figure 1a–c), while 21 peach accessions with rarely observed pollen grains were classified as sterile (Figure 1d–f).

### 2.2. Candidate Gene Identification via GWAS

Approximately 378.5 Gb of clean sequencing reads were generated from the 201 peach accessions, with an average depth of 5.3× and an average coverage of 78.1% (Table S1). We identified a total of 1,042,687 high-quality SNPs among the accessions for the subsequent GWAS analysis.

**Figure 2:**
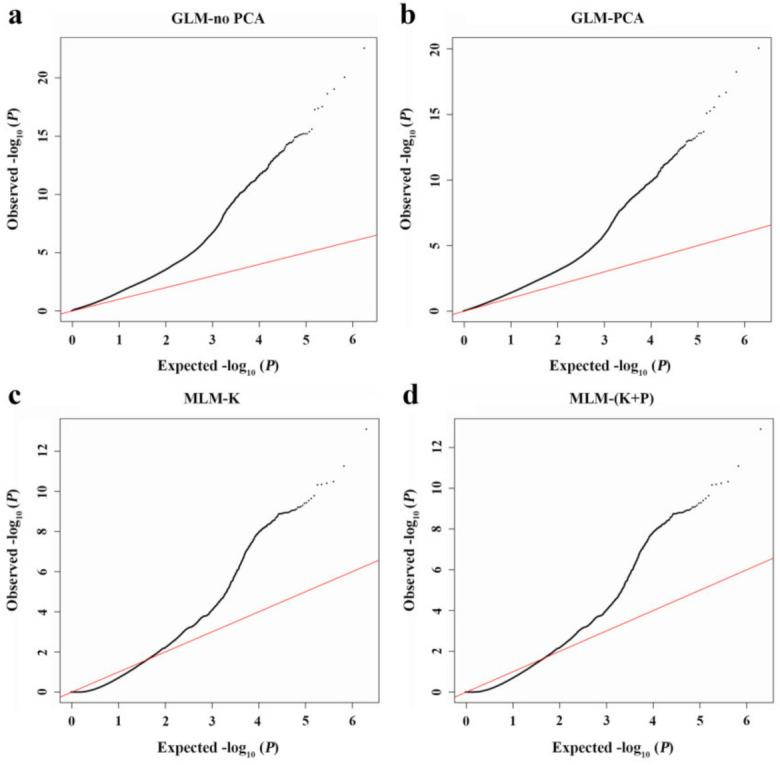
Quantile–quantile (Q–Q) plot of the four analysis models used in the genome-wide association study (GWAS) for peach pollen fertility trait. The red line indicates the expected line under the null distribution. The black line represents the *P* values observed using the (**a**) GLM-no PCA, (**b**) GLM-PCA, (**c**) MLM-K, and (**d**) MLM-(K+P) models, respectively.

To determine the most suitable model for peach pollen fertility trait analysis, we made a comparison between four models: (1) the general linear model without any consideration of principal component analysis (GLM-no PCA); (2) the GLM that took PCA results into account as the fixed effect (GLM-PCA); (3) the mixed linear model that incorporated kinship value (MLM-K); and (4) the MLM using the PCA results and the kinship value as a correction for population structure (MLM-K+P). As shown in Figure 2, the quantile–quantile (Q–Q) plot for the GLM-no PCA model exhibited the highest deviation from the line of expected *P* values versus observed ones, while the GLM-PCA model produced a distribution of *P* values more comparable to the theoretical one; the two MLM models were better than the two GLM models in this regard but exhibited excessive correction. As such, the GLM-PCA model was adopted in our study.

**Figure 3:**
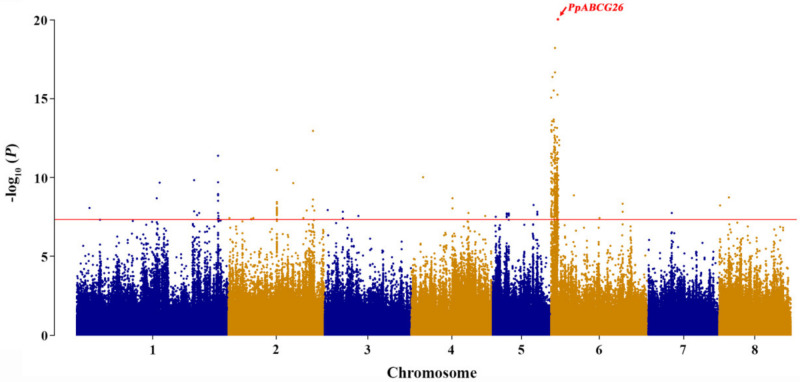
Genome-wide association study result of peach pollen fertility trait. The *y*-axis indicates the association value of the SNPs, and the *x*-axis indicates their positions on the chromosomes. The horizontal red line represents the significance threshold (7.32) for the association of peach pollen fertility trait.

The additional association analysis helped us determine the reasonable loci related to peach pollen fertility (Figure 3). As revealed by the Q–Q plot and Manhattan plots, a significant association peak was located on Chr.06: 2,116,368 bp, with a –log_10_ (*P*) of 19.8. Considering that the LD decay was about 20–50 kb for the different subgroups of cultivated peach [30], we subsequently performed candidate gene screening in the region that covered ±25 kb on either side of the significant association peak. This allowed us to identify nine candidate genes potentially responsible for the peach pollen fertility trait (Table 1).

### 2.3. Candidate Gene Expression Analysis

**Figure 4:**
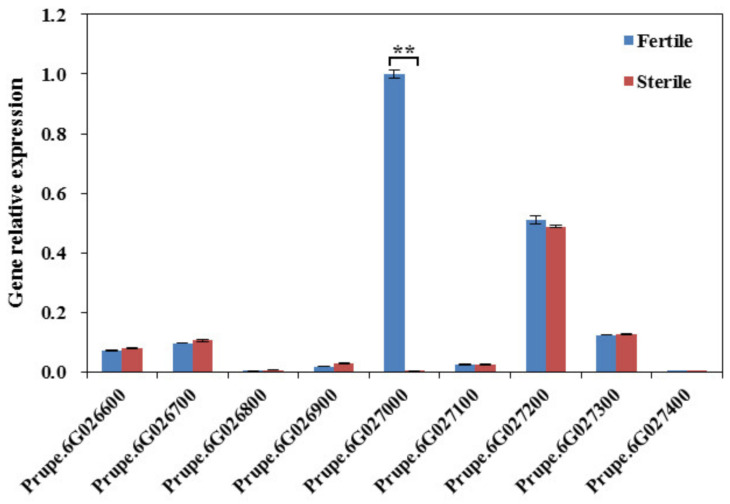
Expression patterns of the nine candidate genes related to peach pollen fertility. The blue bars and red bars indicate the relative expression in the anther samples of the fertile cultivar ‘Da Hong Pao’ and sterile cultivar ‘Annong Shui Mi’, respectively. ** *p* < 0.01.

We measured the expression profiles of the nine candidate genes related to peach pollen fertility in the anther samples from the fertile cultivar ‘Da Hong Pao’ and the sterile cultivar ‘Annong Shui Mi’ (Figure 4). Except for the differentially expressed gene *Prupe.6G027000*, the other eight candidate genes exhibited either no expression in peach anther or undifferentiated expression between fertile and sterile peach anthers. Combined with the functional annotation of gene *Prupe.6G027000* as an ABC transporter G family member 26—the homologs of which have been proven to play a crucial role in pollen grain development in rice and *Arabidopsis*, as mentioned above—here we took said gene as a strong candidate in peach fertility determination and designated it as *PpABCG26*.

### 2.4. Candidate Gene Sequence Analysis

**Figure 5:**
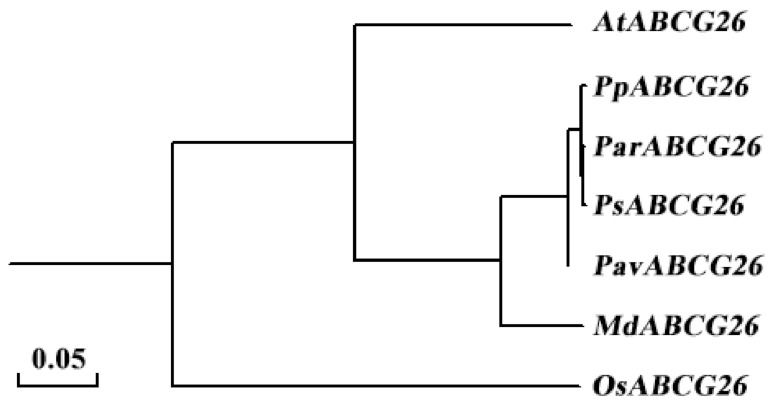
Phylogenetic tree of the ABCG26 proteins of different plant species. The species, from top to bottom, are *Arabidopsis* (*Arabidopsis thaliana*), peach (*Prunus persica*), apricot (*Prunus armeniaca*), Chinese plum (*Prunus salicina*), sweet cherry (*Prunus avium*), apple (*Malus domestica*), and rice (*Oryza sativa*).

**Figure 6:**
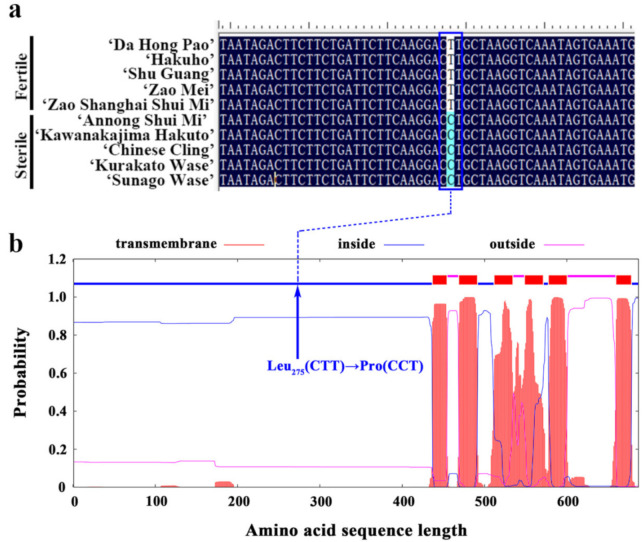
Sequence analysis of the candidate gene *PpABCG26*. (**a**) The sequence alignment of *PpABCG26* in the five fertile and five sterile peach cultivars. The blue box indicates the nonsynonymous SNP (T/C) at +824 bp in the coding region. (**b**) The predicted transmembrane helices of PpABCG26. The blue arrow indicates the resultant amino acid change from Leu_275_ (CTT) to Pro (CCT).

The amino acid sequences of the ABCG26 proteins of Arabidopsis (*Arabidopsis thaliana*), peach (*Prunus persica*), apricot (*Prunus armeniaca*), Chinese plum (*Prunus salicina*), sweet cherry (*Prunus avium*), apple (*Malus domestica*), and rice (*Oryza sativa*) were retrieved from The Arabidopsis Information Resource database (TAIR; https://www.arabidopsis.org/), the Genome Database of Rosaceae (GDR; https://www.rosaceae.org/), and the China Rice Data Center (http://www.ricedata.cn/), and were then subjected to sequence alignment and phylogenetic analysis, resulting in a sequence identity of 81.88% across different species (Figure 5, Figure S1). As revealed by the GWAS results, the significant association peak was at Chr.06: 2,116,368 bp, which displayed polymorphism (T/C) among the 201 peach accessions and was precisely located in the coding sequence (+824 bp) of *PpABCG26*. We then validated this SNP (T/C) using Sanger sequencing in ten randomly selected peach cultivars (five fertile and five sterile). As shown in Figure 6a, the T genotype and C genotype were tightly associated with fertile and sterile phenotypes, respectively. Amino acid translation revealed a transition from Leucine (codon CTT) to Proline (codon CCT). Additionally, using TMHMM Server v. 2.0 (http://www.cbs.dtu.dk/services/TMHMM/), we performed a prediction of transmembrane helices in the PpABCG26 protein (Figure 6b) and found that the resultant amino acid change from Leu_275_ to Pro was predicted to be located on the intracellular binding domain instead of the six transmembrane domains. This suggests that the nonsynonymous SNP (T/C) possibly affected the binding between the transporter and pollen grain development-related substrates and finally led to pollen sterility in peach.

## 3. Discussion

Discovering the genetic factors underlying important agronomic traits continues to draw breeders’ attention since it could improve genomic selection efficiency by providing valuable markers. In the past few decades, for the peach pollen fertility/sterility trait, the top region of Chr.06 (0–7.52 Mb) was characterized as a hotspot by using genetic mapping in different hybrid populations, but it remained difficult to focus on specific genes as the SSR markers used were employed at a low density [24,25,26,27]. With the capabilities of sequencing technologies, high-density molecular markers have been widely adopted in genetic basis dissection [30,31,32,33,34,35,36]. In our study, using the high-quality SNPs generated from the re-sequencing data of 201 peach accessions, a significant association peak was detected at Chr.06: 2,116,368 bp (Figure 3), which is in accordance with the previous genetic mapping results but displays largely improved precision, allowing follow-up candidate gene identification (Table 1).

Combining the results of GWAS, functional annotation, sequence alignment, gene relative expression analysis, and SNP validation, the gene *PpABCG26* was speculated to be responsible for pollen fertility/sterility in peach (Figure 3, Figure 4, and Figure 6). Through evolution, ABCGs have emerged as one of the most conserved but also divergent proteins that safeguard the formation of the male gametophyte via mediating the lipid metabolism and, in particular, the transport of the lipidic and phenolic precursors across both sides of the anther layers, to form the two most important protective barriers of pollen development [38]. By performing sequence alignment and phylogenetic analysis, we found a sequence identity of 81.88% across different flowering species belonging to both *Monocotyledoneae* and *Dicotyledoneae* (Figure 5, Figure S1). As a membrane protein, the critical role of ABCG26 in exine formation and pollen development has been well studied in *Arabidopsis* and rice and is consistent with the model by which ABCG26 transports sporopollenin precursors across the tapetum plasma membrane into the locule for polymerization on developing microspore walls, both of which are plant-specific structures critical to pollen development [16,17,18,19,20].

By genotyping the gene-contained SNP (T/C) in five fertile and five sterile peach cultivars, the T genotype and the C genotype were shown to be tightly associated with the fertile and sterile phenotypes, respectively (Figure 6a). However, by analyzing the re-sequencing data of the 201 peach accessions, we noticed that, for the 21 sterile accessions, 9 of them displayed the heterozygous T/C or homozygous T/T genotype, which was inconsistent with the theological homozygous C/C genotype for sterile cultivars; as regards the 180 fertile accessions, all of them displayed the heterozygous T/C or homozygous T/T genotype, which was in accordance with the theological ones (Table S1). It would be reasonable to assume that, besides the ABCG26 transporter, some other factors that work collaboratively to transport multiple precursors for cutin, wax, sporopollenin, tryphine, intine, and nexine formation may lead to a similar sterile phenotype. In *Arabidopsis*, tapetal cells were shown to generate precursors for the formation of sexine, tryphine, and nexine, which are transferred to other locules by AtABCG26, AtABCG9 and AtABCG31, and AtABCG1 and AtABCG16, crossing the tapetal cells’ plasma membrane, then be transferred to the microspores surface by unknown transporters. AtABCG1 and AtABCG16 also export intine precursors across microspores’ plasma membranes for intine development. In rice, both OsABCG26 and OsABCG15 collaboratively regulate the transport of anther cuticle and sporopollenin precursors; while OsABCG26 mainly transports wax and cutin precursors toward the anther surface for anther cuticle formation, OsABCG15 transport sporopollenin precursors from the tapetum to the anther locule for exine formation [18,37,38]. The natural variation in the coding sequence (+824 bp) of gene *PpABCG26* was a sufficient, but not necessary condition for the peach pollen fertile/sterile phenotype.

## 4. Materials and Methods

### 4.1. Plant Materials

In total, 201 peach accessions were used for whole-genome re-sequencing and subsequent GWAS analysis (Table S1). The fertile cultivar ‘Da Hong Pao’ and the sterile cultivar ‘Annong Shui Mi’ were selected for gene nucleotide sequence analysis and gene relative expression analysis. All the peach accessions were planted at the National Germplasm Repository of Peach in Zhengzhou, Henan, China (35°09′ N, 113°47′ E); these trees were trained to the “Y” system at a density of 2.0 × 5.0 m and were maintained under identical management operations.

### 4.2. Phenotyping

We determined the fertility/sterility of the peach accessions by observing the presence of pollen grains in the naturally dried anthers. The fresh anthers were collected, stored under cool and dry conditions for 72 h, and then directly observed using a microscope [39]. Accessions with abundant pollen grains were classified as fertile (Figure 1a–c), while accessions with rarely observed pollen grains were classified as sterile (Figure 1d–f).

### 4.3. Nucleic Acid Isolation

Genomic DNA was extracted using a QIAGEN^®^ Genomic kit (Qiagen Co., Ltd., Hilden, Germany) from young leaves bursting after blooming, and the total RNA of anther samples was isolated using a Plant Total RNA Extraction Kit (Huayueyang Bio Technology Co., Ltd., Beijing, China), deferring to the manufacturer’s instructions. The quality and quantity of the isolated DNA and RNA were separately checked via electrophoresis on a 0.75% agarose gel and a NanoDrop™ D-1000 spectrophotometer (NanoDrop Technologies, Wilmington, DE, USA).

### 4.4. DNA Sequencing and Association Study

The whole-genome re-sequencing of the 201 peach accessions was completed in our previous work [34]. The library insert size was 500 bp, and the pair-end read size was 150 bp. All libraries were sequenced using the Illumina Hiseq™ X-Ten platform (Illumina, San Diego, CA, USA) by Annoroad Gene Technology (Beijing, China). The qualified paired-end reads of each accession were aligned against the peach reference genome v2.0 [40] using BWA v0.7.12-r1039 [41], and SNPs were identified using the Genome Analysis Toolkit (GATK) [42]. The SNPs with missing data ≥ 20% and minor allele frequencies (MAF) < 2% in the population were discarded, leaving 1,042,687 high-quality SNPs for the following GWAS analysis.

Based on the re-sequencing data of 201 peach accessions, we compared four models with TASSEL v4.1 to determine the best one for our association analysis [43]: the GLM-no PCA, the GLM-PCA, the MLM-k, and the MLM-K+P models. The specific commands to run each model were as follows: (1) GLM-no PCA: ./tassel4.0_standalone/run_pipeline.pl -fork1 -h./genotype.txt -fork2 -r./phenotype.txt -combine4 -input1 -input2 -intersect -glm -export glm_output -runfork1 -runfork2; (2) GLM-PCA: ./tassel4.0_standalone/run_pipeline.pl -fork1 -h./genotype.txt -fork2 -r./phenotype.txt -fork3 -q./PCA.cov -combine5 -input1 -input2 -input3 -intersect -glm -export glm_output -runfork1 -runfork2 -runfork3; (3) MLM-K: ./tassel4.0_standalone/run_pipeline.pl -fork1 -h./genotype.txt -fork2 -r./phenotype.txt -fork3 -k./kinship.txt -combine4 -input1 -input2 -intersect -combine5 -input3 -input4 -mlm -mlmVarCompEst P3D -mlm CompressionLevel Optimum -export mlm_output -runfork1 -runfork2 -runfork3; and (4) MLM-K+P: ./tassel4.0_standalone/run_pipeline.pl -fork1 -h./genotype.txt -fork2 -r./phenotype.txt -fork3 -q./PCA.cov -fork4 -k./kinship.txt -combine5 -input1 -input2 -input3 -intersect -combine6 -input5 -input4 -mlm -mlmVarCompEst P3D -mlmCompressionLevel None -export mlm_output -runfork1 -runfork2 -runfork3 -runfork4 [32].

A stringent Bonferroni correction was used to screen obvious association signals based on the *P* value, calculated by dividing 0.05 by the number of SNPs. Manhattan and quantile–quantile (Q–Q) plots were generated in R v3.5.1 using the package qqman v0.1.2 [44].

### 4.5. Candidate Gene Sequence Analysis

The reference nucleotide sequences of candidate genes were obtained from the Genome Database of Rosaceae (GDR; https://www.rosaceae.org/). The candidate gene-specific primers (Sense: 5′ CATCAAAGGCATATCAGG 3′; Anti-sense: 5′ TTGTCCAGTGGCTAAATC 3′) were designed using the Primer-BLAST tool integrated with the National Center for Biotechnology Information Database (NCBI) (https://www.ncbi.nlm.nih.gov/tools/primer-blast/). The PCRs were performed via procedures set according to the product lengths and primer annealing temperatures. The PCR products comprising SNP loci of interest were then subjected to Sanger sequencing at Sangon Biotech (Shanghai, China) Co., Ltd.

### 4.6. Candidate Gene Expression Analysis

The fresh anther samples of the fertile cultivar ‘Da Hong Pao’ and the sterile cultivar ‘Annong Shui Mi’ were collected every three days from 1 March to 15 April in 2019, corresponding with the period from the sporogenous cell formation stage to the balloon-flower stage [45]. The anther samples of the two cultivars were mixed respectively and then subjected to RNA isolation. First-strand cDNA synthesis was carried out using 1.0 mg qualified RNA and the Transcriptor First Strand cDNA synthesis kit (Takara, Dalian, China) according to the manufacturer’s instructions. The gene-specific primers were also designed using the Primer-BLAST tool (Table S2). The qRT-PCRs were performed using the LightCycler System (Roche LightCycler 480, Roche Diagnostics, Basel, Switzerland) following the manufacturer’s instructions. The gene relative expression levels were calculated with the 2^−ΔΔCT^ method.

## 5. Conclusions

By conducting GWAS on 201 peach accessions, we detected a significant association peak for the peach pollen fertility trait at Chr.06: 2,116,368 bp, which was located in the coding region of the gene *PpABCG26*. Based on the results of the gene function comparison, gene relative expression, and nucleotide sequence analysis, *PpABCG26* was proposed as a key factor in controlling peach pollen fertility/sterility. These results will help us to understand the genetic basis of the peach pollen fertility trait and to discover applicable markers for pre-selection in peach.

## Figures and Tables

**Table 1:** Information pertaining to candidate genes for peach pollen fertility.

Gene ID	Physical Position (bp)	Functional Annotation
*Prupe.6G026600*	Chr.06: 2,092,736–2,096,757	Heterogeneous nuclear ribonucleoprotein 1
*Prupe.6G026700*	Chr.06: 2,100,008–2,106,576	Kinase-related protein of unknown function
*Prupe.6G026800*	Chr.06: 2,106,907–2,110,035	Nucleotide/sugar transporter family protein
*Prupe.6G026900*	Chr.06: 2,111,266–2,114,599	Coiled-coil domain-containing protein 22
*Prupe.6G027000*	Chr.06: 2,115,131–2,118,488	ABC transporter G family member 26
*Prupe.6G027100*	Chr.06: 2,118,854–2,122,002	Transmembrane protein 245
*Prupe.6G027200*	Chr.06: 2,122,159–2,125,309	Protein CWC15 homolog A
*Prupe.6G027300*	Chr.06: 2,132,488–2,134,587	Viral IAP-associated factor homolog
*Prupe.6G027400*	Chr.06: 2,135,267–2,145,546	Mediator of RNA polymerase II transcription subunit 15a

Note: Bp, basepair. Chr, chromosome.

## Data Availability

The referenced SNPs have also been deposited in the Figshare database (https://figshare.com/s/7e177dad8e036742c0fb).

## Supplementary Materials

Figure S1. Sequence alignments of ABCG26 proteins of Arabidopsis (*Arabidopsis thaliana*), peach (*Prunus persica*), apricot (*Prunus armeniaca*), Chinese plum (*Prunus salicina*), sweet cherry (*Prunus avium*), apple (*Malus domestica*), and rice (*Oryza sativa*). Table S1. Sequencing information of the 201 peach accessions used for GWAS analysis. Table S2. Gene-specific primers for qRT-PCR.
