# Supplementary figures and images for: Preliminary Identification of Key Genes Controlling Peach Pollen Fertility Using Genome-Wide Association Study
Source: Plants (Basel). 2021 Jan 27;10(2):242. doi: 10.3390/plants10020242 (PMC7911534; doi:10.3390/plants10020242)

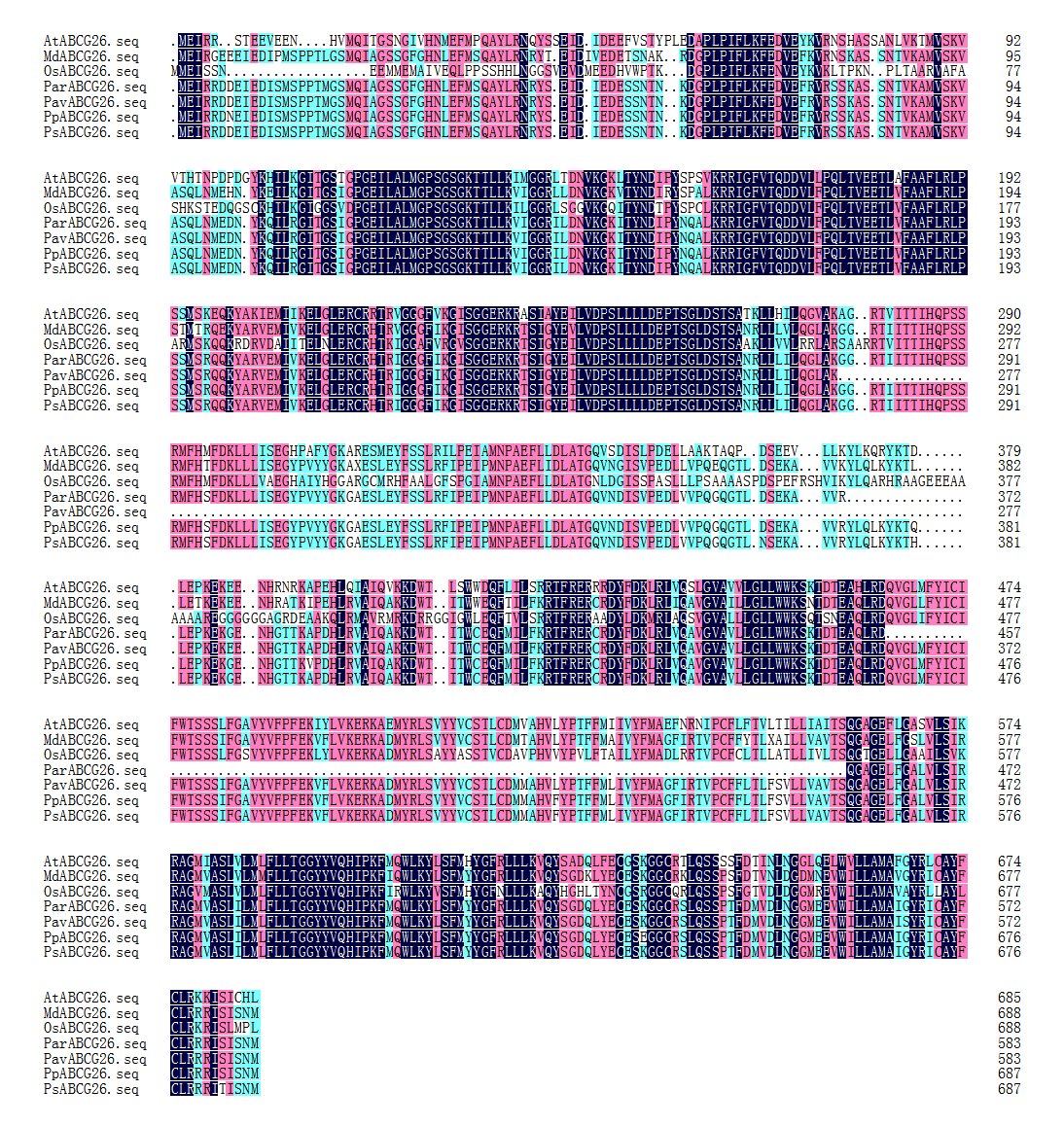

Supplement: Supplementary file 1 [file plants-10-00242-s001.zip › Figure S1 Sequence alignments of ABCG26 proteins.tif]
